# Supplementary material for: Psilocybin Attenuates Cortical Representations of Aversion in the Mouse Auditory Cortex
Source: bioRxiv. 2026 Mar 27:2026.03.26.714498. Preprint. [Version 1] doi: 10.64898/2026.03.26.714498 (PMC13041856; doi:10.64898/2026.03.26.714498)
Supplement: Supplement 2 [file media-2.pdf]

Figure	Panel	Outcome	Experimental_condition	Test	Test_statistic	p_value	df	p adjustment & notes	N_mice (psy, sal)	N_cells: psy	N_cells: sal
1	E	Treatment vs session (% of subpopulation)	Tone and stimuli response categories	Compositional Data Analysis	Aitchison Distance	p value		type III Wald chi-square tests from linear mixed-effects models with Mouse as random effect	5, 5		
			Aitchison Distance	Pre (Sal vs PSY)	2.405	0.158				333	521
				Post (Sal vs PSY)	0.831	0.991				229	498
				Chi-square test	Chi-squared	p value			5, 5		
			V1 (Tone-only vs others)	Group	0.067	0.796	1.00				
				Session	1.683	0.195	1.00				
				Group:Session	0.986	0.321	1.00				
			V2 (mixed-balances)	Group	1.953	0.162	1.00				
				Session	0.084	0.772	1.00				
				Group:Session	1.461	0.227	1.00				
			V3 (mixed-balances)	Group	2.635	0.105	1.00				
				Session	1.753	0.185	1.00				
				Group:Session	0.002	0.969	1.00				
			V4 (mixed-balances)	Group	0.001	0.981	1.00				
				Session	0.053	0.819	1.00				
				Group:Session	0.020	0.887	1.00				
			V5 (mixed-balances)	Group	0.315	0.575	1.00				
				Session	1.350	0.245	1.00				
				Group:Session	0.369	0.543	1.00				
			V6 (mixed-balances)	Group	1.809	0.179	1.00				
				Session	18.967	0.00001 ***	1.00				
				Group:Session	1.655	0.198	1.00				
1	H-left	Treatment vs session (Peak spike rate)	Non-paired	Repeated-measures mixed model	F value	p value		Mouse and unique cell as random effects	5, 5	171	137
				Treatment	0.110	0.741	1, 306				
				Session	18.009	2.918e-05 ***	1, 306				
				Treatment x sesson interaction	0.148	0.700	1, 306				
1	H-middle	Treatment vs session (Peak spike rate)	Water-paired	Repeated-measures mixed model	F value	p value		Mouse and unique cell as random effects	5, 5	234	229
				Treatment	1.192	0.307	1, 7.81				
				Session	2.230	0.136	1, 461				
				Treatment x sesson interaction	3.182	0.075	1, 461				
1	H-right	Treatment vs session (Peak spike rate)	Puff-paired	Repeated-measures mixed model	F value	p value		Mouse and unique cell as random effects	5, 5	227	208
				Treatment	0.264	0.621	1, 8				
				Session	14.657	0.000148 ***	1, 433				
				Treatment x sesson interaction	0.019	0.891	1, 433				
1	I-left	Treatment vs session (Peak spike rate)	Water	Repeated-measures mixed model	F value	p value		Mouse and unique cell as random effects	5, 5	119	135
				Treatment	0.002	0.969	1, 7.49				
				Session	0.008	0.929	1, 252				
				Treatment x sesison interaction	5.746	0.01726 *	1, 252				
				Post-hoc comparisons: Holm-Bonferroni				Mouse and unique cell as random effects			
				Psy: Pre vs Post	-1.583	0.115	252.00				
				Sal: Pre vs Post	1.816	0.071	252.00				
1	I-right	Treatment vs session (Peak spike rate)	Air puff	Repeated-measures mixed model	F value	p value		Mouse and unique cell as random effects	5, 5	274	224
				Treatment	2.447	0.155	1, 8.3				
				Session	10.780	0.00100 **	1, 496				
				Treatment x sesson interaction	10.912	0.00103 **	1, 496				
				Post-hoc comparisons: Holm-Bonferroni				Mouse and unique cell as random effects			
				Psy: Pre vs Post	4.910	<0.0001	496.00				
				Sal: Pre vs Post	-0.013	0.989	496.00				
1	J	Session per Treatment (Peak spike rate)	Tones: Water-paired/air puff-paired	3-way repeated-measures mixed model	F value	p value		Mouse and unique cell as random effects	5, 5	1189	1261
				Water-paired:airpuff-paired vs treatment vs session	0.358	0.550	1, 4892				
				Linear regression analysis: Psilocybin	39.710	<0.0001	2, 3561				
				Linear regression analysis: Saline	2.047	0.129	2, 3777				
1	K	Session per Treatment (Peak spike rate)	Stimuli: Water/air puff	3-way repeated-measures mixed model	F value	p value		Mouse and unique cell as random effects	5, 5	1189	1261
				Water:airpuff vs treatment vs session	4.657	0.031 *	1, 4892				
				Linear regression analysis: Psilocybin	7.151	0.0008 **	2, 3561				
				Linear regression analysis: Saline	1.105	0.331	2, 3777				
2	C	Tone preference (% of subpopulation)	Treatment vs session vs prefered tone %	Compositional Data Analysis	Chi-squared	p value		Type III Wald chi-square tests from linear mixed-effects models with Mouse as random effec		912, 795, 984, 280	615, 601, 657, 520
			V1 (Tone 1 vs Others)	Session	0.083	0.773	1.00		13,10		
				Tone	3.371	0.338	1.00				
				Group × Session	3.371	0.338	3.00				
			V2 (Tone 2 vs Tone 3)	Session	0.031	0.860	1.00		13,10		
				Tone	2.448	0.485	3.00				
				Group × Session	2.448	0.485	3.00				
2	D	Treatment vs session (% of population)	All tones	Repeated-measures mixed model	F value	p value		Mouse and unique cell as random effects	13,10	2450	1699
				Treatment	1.081	0.310	1,20.861				
				Session	3.764	0.01557 *	3, 56.616				
				Treatment x sesson interaction	0.268	0.848	3, 56.616				
2	E	Treatment vs session (% of population)	Tone 1 (Water-paired)	Repeated-measures mixed model	F value	p value		Mouse and unique cell as random effects	13,10	294, 240, 286, 81	206, 188, 208, 160
				Treatment	1.997	0.172	1, 21.216				
				Session	2.982	0.0388 *	3, 56.616				
				Treatment x sesson interaction	0.010	0.999	3, 56.616				
2	E	Treatment vs session (% of population)	Tone 2 (Non-paired)	Repeated-measures mixed model	F value	p value		Mouse and unique cell as random effects	13,10	382, 334, 464, 129	250, 270, 297, 240
				Treatment	1.860	0.187	1, 21.821				
				Session	3.936	0.0127 *	3, 57.546				
				Treatment x sesson interaction	0.204	0.893	3, 57.546				
2	E	Treatment vs session (% of population)	Tone 3 (Puff-paired)	Repeated-measures mixed model	F value	p value		Mouse and unique cell as random effects	13,10	236, 221, 234, 70	159, 143, 152, 120
				Treatment	0.719	0.406	1, 20.9				
				Session	1.197	0.319	3, 56.549				
				Treatment x sesson interaction	0.094	0.963	3, 56.549				
2	G	Treatment vs session (Peak spike rate)	Tone 1 (Water-paired)	Repeated-measures mixed model	F value	p value		Mouse and unique cell as random effects	13,10	294, 240, 286, 81	206, 188, 208, 160
				Treatment	2.575	0.124	20.71				
				Session	4.576	0.00339 **	1425.85				
				Treatment x sesson interaction	3.659	0.0120 *	1425.85				
				Post-hoc comparisons: Holm-Bonferroni	t-ratio	p value					
				Pre: Psy vs Sal	0.418	0.678	39.60				
				Post: Psy vs Sal	-2.246	0.0297 *	44.30				
				Post +1: Psy vs Sal	-1.077	0.288	39.70				
				Post +8: Psy vs Sal	-2.006	0.0478 *	93.10				
2	H-left	Post session (Cumulative distribution)	Tone 1 (Water-paired)	Kolmogorov–Smirnov test: Post	D = 0.275	0.0000380 **			13,10	240, 188	
2	H-right	Post +8 session (Cumulative distribution)	Tone 1 (Water-paired)	Kolmogorov–Smirnov test: Post +8	D = 0.278	0.00154 **			13,10	81, 160	
2	I	Treatment vs session (Peak spike rate)	Tone 2 (Non-paired)	Repeated-measures mixed model	F value	p value		Mouse and unique cell as random effects	13,10	382, 334, 464, 129	250, 270, 297, 240
				Treatment	6.792	0.0165 *	21.02				
				Session	2.752	0.0413 *	2006.73				
				Treatment x sesson interaction	0.184	0.907	2006.73				
2	I	Treatment vs session (Normalized to Pre)	Tone 2 (Non-paired)	Repeated-measures mixed model	F value	p value		Mouse-level	13,10		
				Treatment	0.998	0.329	1, 21				
				Session	3.098	0.0339 *	3, 56				
				Session x Treatment	0.635	0.596	3, 56				
2	J	Treatment vs session (Peak spike rate)	Tone 3 (Puff-paired)	Repeated-measures mixed model	F value	p value		Mouse and unique cell as random effects	13,10	236, 221, 234, 70	159, 143, 152, 120
				Treatment	1.725	0.202	22.52				
				Session	4.473	0.00393 **	1225.07				
				Treatment x sesson interaction	0.906	0.438	1225.07				
2	K	Treatment vs Post trial stages (Peak spike rate)	All tones	Repeated-measures mixed model	F value	p value		Geisser-Greenhouse's	13,10		
			Paired tone	Trial stage	108.000	<0.0001	1.597, 31.95				
				Group	2.055	0.167	1,20				
				Trial stage x Group	1.267	0.293	2,40				
			Unpaired tones	Trial stage	44.510	<0.0001	1.914, 40.19				
				Group	4.288	0.051	1,20				
				Trial stage x Group	0.288	0.751	2,40				
2	L-left	Treatment vs session (Noise correlation)	Tone 1 (Water-paired)	Repeated-measures mixed model	F value	p value		Mouse level	11,10	2161	1699
				Treatment	0.012	0.913	1, 20				
				Session	0.339	0.742	2.275, 39.43				
				Treatment x sesson interaction	3.789	0.0265 *	2.275, 39.43				
				Post-hoc comparisons: Holm-Bonferroni	t-ratio	p value		Mouse level			
				Pre: Psy vs Sal	0.054	>0.9999	14.22				
				Post: Psy vs Sal	1.711	0.356	17.97				
				Post +1: Psy vs Sal	2.968	0.0393 *	14.32				
				Post +8: Psy vs Sal	0.209	0.999	11.95				
2	L-middle	Treatment vs session (Noise correlation)	Tone 2 (Non-paired)	Repeated-measures mixed model	F value	p value		Mouse level	11,10	2161	1699
				Treatment	5.727	0.417	1, 19				
				Session	0.690	0.572	2.588, 44.86				
				Treatment x sesison interaction	1.783	0.171	2.588, 44.86				
2	L-right	Treatment vs session (Noise correlation)	Tone 3 (Puff-paired)	Repeated-measures mixed model	F value	p value		Mouse level	11,10	2161	1699
				Treatment	1.087	0.309	1, 19				
				Session	3.858	0.0225 *	2.357, 45.58				
				Treatment x sesison interaction	0.640	0.201	2.357, 45.58				
2	M	Treatment vs session (Noise correlation)	All tones	Repeated-measures mixed model	F value	p value		Mouse level	11,10	2161	1699
				Treatment	1.027	0.324	1, 19				
				Session	1.033	0.376	2.411, 41.79				
				Treatment x sesison interaction	5.682	0.00190 **	2.411, 41.79				
				Post-hoc comparisons: Sidak	t-ratio			Mouse level			
				Pre: Psy vs Sal	0.275	0.998	18.68				
				Post: Psy vs Sal	2.893	0.0381 *	18.02				
				Post +1: Psy vs Sal	1.549	0.461	14.45				
				Post +8: Psy vs Sal	0.907	0.852	13.93				
3	C	Treatment vs session (Peak spike rate)	Air puff	Repeated-measures mixed model	F value	p value		Mouse level	13,10	414, 446, 368, 86	236, 250, 262, 229
				Treatment	2.931	0.102	1, 21.14				
				Session	23.148	1.108e-14 ***	3, 1679.27				
				Treatment x sesson interaction	3.798	0.00990 **	3, 1679.27				
				Post-hoc comparisons: Holm-Bonferroni	t-ratio	p value		Mouse level	13,10		
				Pre: Psy vs Sal	-0.005	0.996	31.40				
				Post: Psy vs Sal	-2.062	0.0478 *	30.40				
				Post +1: Psy vs Sal	-1.667	0.106	31.00				
				Post +8: Psy vs Sal	-2.131	0.0371 *	62.00				
3	D-left	Post session (Cumulative distribution)	Air puff	Kolmogorov–Smirnov test: Post	D = 0.143	0.0420 *			13,10	446	250
3	D-right	Post +8 session (Cumulative distribution)	Air puff	Kolmogorov–Smirnov test: Post +8	D = 0.137	0.306			13,10	86	229
3	E	Treatment vs Post trial stages (Peak spike rate)	Air puff	Repeated-measures mixed model	F value	p value		Mouse level	13,10	446	250
				Trial stage	47.450	< 0.0001	1.11, 23.33	Geisser-Greenhouse's			
				Treatment	0.048	0.829	1,21				
				Trial stage x Treatment	0.403	0.671	2, 42				
3	F	Treatment vs session (% of population)	Air puff	Repeated-measures mixed model				Mouse level	13,10		
				Treatment	0.418	0.525	1, 21	Geisser-Greenhouse's		414, 446, 368, 86	236, 250, 262, 229
				Session	3.542	0.0280 *	2.493, 46.54				
				Treatment x sesson interaction	2.186	0.100	3, 56				
3	G	Treatment vs session (Noise correlation)	Air puff	Repeated-measures mixed model	F value	p value		Mouse level	13,10	2450	1699
				Treatment	0.3425	0.565	1, 20				
				Session	0.382	0.262	2.127, 42.53				
				Treatment x sesson interaction	1.197	0.319	3,60				
4	B	Treatment vs session (% of subpopulation)	Tone and stimuli response categories	Compositional Data Analysis	Chi-squared	p value		Type III Wald chi-square tests from linear mixed-effects models with Mouse as random effect		930, 951, 968, 291	567, 560, 594, 524
			V1 (Tone-only vs Others)	Session	0.108	0.742	3.00		13,10		
				Group	6.905	0.075	1.00				
				Group × Session	10.377	0.0160 *	3.00				
			V1 (Tone-only vs Others)	Post-hoc comparisons: Bonferroni	t-ratio	p value					
				Pre: Psy vs Sal	0.270	0.188	87.10				
				Post: Psy vs Sal	0.225	0.271	87.10				
				Post +1: Psy vs Sal	-0.112	0.583	87.10				
				Post +8: Psy vs Sal	-0.545	0.0309 *	87.10				
			V2 (Air puff-only vs Tone & air puff)	Session	4.423	0.0354 *	3.00		13,10		
				Group	9.775	0.0205 *	1.00				
				Group × Session	1.939	0.585	3.00				
4	C	Treatment vs session (Peak spike rate)	Tone only (Tone block)	Repeated-measures mixed model	F value	p value		Mouse and unique cell as random effects	13,10	516, 505, 600, 205	331, 310, 332, 295
				Treatment	1.737	0.585	1, 21.21				
				Session	6.205	0.000338 ***	3, 2608.80				
				Treatment x sesson interaction	0.869	0.456	3, 2608.80				
4	D	Treatment vs session (Peak spike rate)	Tone & air puff (Tone block)	Repeated-measures mixed model	F value	p value		Mouse and unique cell as random effects	13,10	175, 135, 164, 31	128, 123, 135, 105
				Treatment	1.737	0.378	1, 19.76				
				Session	2.179	0.089	3, 694.74				
				Treatment x sesson interaction	0.708	0.547	3, 694.74				
4	E	Treatment vs session (Peak spike rate)	Air puff only (Air puff block)	Repeated-measures mixed model	F value	p value		Mouse and unique cell as random effects	13,10	239, 311, 204, 55	108, 127, 127, 124
				Treatment	1.270	0.272	1, 22.22				
				Session	9.487	3.509e-06 ***	3, 987.73				
				Treatment x sesson interaction	4.145	0.0062 **	3, 987.73				
				Post-hoc comparisons: Holm-Bonferroni	t-ratio	p value					
				Pre: Psy vs Sal	0.872	0.388	43.50				
				Post: Psy vs Sal	-0.595	0.556	37.40				
				Post +1: Psy vs Sal	-1.168	0.250	40.90				
				Post +8: Psy vs Sal	-2.454	0.0161 *	86.60				
4	F	Treatment vs session (Peak spike rate)	Air puff & tone (Air puff block)	Repeated-measures mixed model	F value	p value		Mouse and unique cell as random effects	13,10	175, 135, 164, 31	128, 123, 135, 105
				Treatment	2.633	0.120	1, 21.10				
				Session	13.868	8.518e-09 ***	3, 710.05				
				Treatment x sesson interaction	1.075	0.359	3, 710.05				

Figure	Panel	Outcome	Experimental_condition	Test	Test statistic	p value	df	p adjustment & notes	N mice (psy, sal)	N_cells: psy	N_cells: sal
**S1**	**B**	**Treatment vs session (SNR)**	**Tones and air puff**	**Repeated-measures mixed model**	**F value**	**p value**		Geisser-Greenhouse	13, 10		
				Treatment	0.034	0.856	1,21				
				Session	1.201	0.311	1.944, 37.81				
				Treatment x sesson interaction	0.902	0.525	9, 175				
**S1**	**C**	**Treatment vs session (Laser Power)**	**Tones and air puff**	**Repeated-measures mixed model**	**F value**	**p value**		Geisser-Greenhouse	13, 10		
**S3**	**B-left**	**Male vs female, vs session**	**All tones: Male vs female**	**Paired t-tests**	**t value**	**p value**		Mouse level	5, 5		
				Pre Psy: Male vs female	0.747	0.509	3		3, 2		
				Pre Sal: Male vs female	-1.209	0.313	3		3, 2		
				Post Psy: Male vs female	0.918	0.426	3		2, 3		
				Post Sal: Male vs female	-0.293	0.789	3		2, 3		
**S3**	**B-right**	**Male vs female, vs session**	**Stimuli: Male vs female**	**Paired t-tests**	**t value**	**p value**		Mouse level	5, 5		
				Pre Psy: Male vs female	1.549	0.219	3		3, 2		
				Pre Sal: Male vs female	-1.149	0.334	3		3, 2		
				Post Psy: Male vs female	3.881	0.0300 *	3		2, 3		
				Post Sal: Male vs female	-1.342	0.272	3		2, 3		
**S3**	**C-left**	**Treatment vs session (Peak spike rate)**	**Non-paired**	**Repeated-measures mixed model**	**F value**	**p value**		Mouse level, mouse as random effect	5, 5		
				Treatment	0.182	0.680	1, 8.5				
				Session	5.572	0.0459 *	1, 8				
				Treatment x sesson interaction	0.332	0.580	1, 8				
**S3**	**C-middle**	**Treatment vs session (Peak spike rate)**	**Water-paired**	**Repeated-measures mixed model**	**F value**	**p value**		Mouse level, mouse as random effect	5, 5		
				Treatment	0.645	0.439	1, 11.2				
				Session	0.087	0.776	1, 8				
				Treatment x sesson interaction	0.153	0.706	1, 8				
**S3**	**C-right**	**Treatment vs session (Peak spike rate)**	**Puff-paired**	**Repeated-measures mixed model**	**F value**	**p value**		Mouse level, mouse as random effect	5, 5		
				Treatment	0.277	0.610	1, 10.6				
				Session	2.536	0.150	1, 8				
				Treatment x sesson interaction	0.093	0.768	1, 8				
**S3**	**D-left**	**Treatment vs session (Peak spike rate)**	**Water**	**Repeated-measures mixed model**	**F value**	**p value**		Mouse level, mouse as random effect	5, 5		
				Treatment	2.034	0.190	1, 8.36				
				Session	0.053	0.823	1, 8				
				Treatment x sesson interaction	2.098	0.186	1, 8				
**S3**	**D-right**	**Treatment vs session (Peak spike rate)**	**Air puff**	**Repeated-measures mixed model**	**F value**	**p value**		Mouse level, mouse as random effect	5, 5		
				Treatment	1.500	0.254	1, 8.46				
				Session	2.341	0.165	1, 8				
				Treatment x sesson interaction	0.926	0.364	1, 8				
**S3**	**E-left**	**Treatment vs session (Peak spike rate)**	**Non-evoked**	**Repeated-measures mixed model**	**F value**	**p value**		Cell-level, mouse and unique cell as random effects	5, 5	1189	1261
				Treatment	1.842	0.176	1, 397.4				
				Session	2.274	0.132	1, 2448				
				Treatment x sesson interaction	3.632	0.057	1, 2448				
**S3**	**E-right**	**Treatment vs session (Peak spike rate)**	**Non-evoked**	**Repeated-measures mixed model**	**F value**	**p value**		Mouse level, mouse as random effect	5, 5		
				Treatment	1.895	0.188	1, 16				
				Session	0.001	0.981	1, 16				
				Treatment x sesson interaction	2.147	0.162	1, 16				
**S4**	**A**	**Treatment vs session (Peak spike rate)**	**Tone 1 (Previously-paired)**	**Repeated-measures mixed model**	**F value**	**p value**		Mouse level, mouse as random effect	13, 10		
				Treatment	0.723	0.405	1, 21.3				
				Session	1.748	0.168	3,57				
				Treatment x sesson interaction	0.943	0.426	3,57				
**S4**	**B**	**Treatment vs session (Peak spike rate)**	**Tone 2 (Non-paired)**	**Repeated-measures mixed model**	**F value**	**p value**		Mouse level, mouse as random effect	13, 10		
				Treatment	4.508	0.0456 *	1, 21.3				
				Session	3.153	0.0317 *	3,57				
				Treatment x sesson interaction	0.161	0.922	3,57				
				**Post-hoc comparisons: Holm-Bonferroni**	**t value**	**p value**					
				Pre: Psy vs Sal	-1.349	0.183	53.2				
				Post: Psy vs Sal	-1.602	0.115	53.2				
				Post +1: Psy vs Sal	-2.061	0.0442 *	53.2				
				Post +8: Psy vs Sal	-1.235	0.221	67.5				
**S4**	**C**	**Treatment vs session (Peak spike rate)**	**Tone 3 (Post-paired)**	**Repeated-measures mixed model**	**F value**	**p value**		Mouse level, mouse as random effect	13, 10		
				Treatment	1.942	0.178	1, 21.7				
				Session	3.342	0.0254 *	3,57				
				Treatment x sesson interaction	1.608	0.198	3,57				
**S4**	**D**	**Treatment vs session (Peak spike rate)**	**Air puff**	**Repeated-measures mixed model**	**F value**	**p value**		Mouse level, mouse as random effect	13, 10		
				Treatment	2.742	0.112	1, 21.6				
				Session	6.243	0.00097 ***	3,57				
				Treatment x sesson interaction	0.700	0.556	3,57				
**S5**	**A-left**	**Male vs female, vs session**	**All tones: Male vs female**	**Repeated-measures mixed model**	**F value**	**p value**		Mouse level	13, 10		
		Psilocybin		Session	0.932	0.382	1.44, 18.72				
				Sex	0.169	0.846	2, 14				
				Sex x sesson interaction	0.909	0.498	6, 39				
**S5**	**A-right**	**Male vs female, vs session**	**All tones: Male vs female**	**Repeated-measures mixed model**	**F value**	**p value**		Mouse level	13, 10		
		Saline		Session	4.479	0.0248 *	2.1, 18.22				
				Sex	1.398	0.265	1,10				
				Sex x sesson interaction	2.082	0.127	3,26				
**S5**	**B-left**	**Male vs female, vs session**	**Air puff: Male vs female**	**Repeated-measures mixed model**	**F value**	**p value**		Mouse level	13, 10		
		Psilocybin		Session	3.252	0.057	1.92, 24.94				
				Sex	0.767	0.483	2,14				
				Sex x sesson interaction	0.609	0.722	6,39				
**S5**	**B-right**	**Male vs female, vs session**	**Air puff: Male vs female**	**Repeated-measures mixed model**	**F value**	**p value**		Mouse level	13, 10		
		Saline		Session	5.270	0.014 *	2.1, 18.56				
**S6**	**A**	**Treatment vs session (Peak spike rate)**	**Non-evoked**	**Repeated-measures 2-way ANOVA**	**F value**	**p value**		Geisser-Greenhouse	13, 10		
				Treatment	7.506	0.0148 *	1, 21				
				Session	3.705	0.068	1, 21				
				Treatment x sesson interaction	2.168	0.156	1, 21				
**S6**	**B**	**Treatment vs session (delta F / F)**	**Tones: Non-evoked**	**Repeated-measures mixed model**	**F value**	**p value**		Geisser-Greenhouse	13, 10		
				Treatment	1.979	0.174	1, 21				
				Session	1.979	0.174	1.12, 20.8				
				Treatment x sesson interaction	0.904	0.445	3, 56				
**S6**	**C**	**Treatment vs session (delta F / F)**	**Air puff: Non-evoked**	**Repeated-measures mixed model**	**F value**	**p value**		Geisser-Greenhouse	13, 10		
				Treatment	3.235	0.168	1, 21				
				Session	2.040	0.083	1.11, 20.7				
				Treatment x sesson interaction	0.106	0.956	3, 56				
**S7**	**B-top**	**Treatment vs session (Sum movement post-onset)**	**Tone 1 (Previously-paired)**	**Repeated-measures mixed model**	**F value**	**p value**		Geisser-Greenhouse	13, 10		
				Treatment	0.127	0.725	1, 21				
				Session	0.876	0.335	0.8, 15.7				
				Treatment x sesson interaction	0.784	0.508	3, 56				
**S7**	**B-middle**	**Treatment vs session (Sum movement post-onset)**	**Tone 2 (Non-paired)**	**Repeated-measures mixed model**	**F value**	**p value**		Geisser-Greenhouse	13, 10		
				Treatment	0.000	0.975	1, 21				
				Session	0.615	0.434	0.8, 15.7				
				Treatment x sesson interaction	0.800	0.499	3, 56				
**S7**	**B-bottom**	**Treatment vs session (Sum movement post-onset)**	**Tone 3 (Post-paired)**	**Repeated-measures mixed model**	**F value**	**p value**		Geisser-Greenhouse	13, 10		
				Treatment	0.099	0.757	1, 21				
				Session	0.623	0.413	0.8, 15.7				
				Treatment x sesson interaction	1.083	0.364	3, 56				
**S7**	**D**	**Treatment vs session (Sum movement post-onset)**	**All tones**	**Repeated-measures mixed model**	**F value**	**p value**		Geisser-Greenhouse	13, 10		
				Treatment	0.056	0.815	1, 21				
				Session	0.719	0.379	0.79, 14.85				
				Treatment x sesson interaction	0.892	0.451	3, 56				
**S7**	**E**	**Treatment vs session (Movement vs Peak spike rate)**	**All tones**	**Pearson correlation**							
			Psilocybin	r	-0.025	0.836					
				r^2	0.001						
			Saline	r	0.106	0.467					
				r^2	0.011						
**S7**	**G**	**Treatment vs session (Sum movement post-onset)**	**Air puff**	**Repeated-measures mixed model**	**F value**	**p value**		Geisser-Greenhouse	13, 10		
				Treatment	0.039	0.845	1, 21				
				Session	2.247	0.093	3, 56				
				Treatment x sesson interaction	2.346	0.083	3, 56				
**S7**	**H**	**Treatment vs session (Movement vs Peak spike rate)**	**Air puff**	**Pearson correlation**							
			Psilocybin	r	-0.155	0.158					
				r^2	0.024						
			Saline	r	-0.089	0.462					
				r^2	0.008						
**S8**	**C**	**Pairing 1 vs pairing 2 (Blink amplitude)**	**Air puff**	**Repeated-measures mixed model**	**F value**	**p value**		Geisser-Greenhouse	6, 10		
				Treatment	0.014	0.908	1, 14				
				Pairing 1 vs pairing 2	0.834	0.377	1, 14				
				Treatment x sesson interaction	0.016	0.900	1, 14				
**S8**	**D**	**Treatment vs session (Blink amplitude)**	**Air puff**	**Repeated-measures mixed model**	**F value**	**p value**		Geisser-Greenhouse	6, 10		
				Treatment	0.218	0.645	1,22				
				Session	0.351	0.762	2.62, 50.65				
				Treatment x sesson interaction	0.964	0.416	3, 58				
**S8**	**E**	**Treatment vs session (Blink amplitude)**	**Tone 1 (Previously-paired)**	**Repeated-measures mixed model**	**F value**	**p value**		Geisser-Greenhouse	13, 11		
				Treatment	0.758	0.393	1,22				
				Session	1.218	0.311	2.613, 50.52				
				Treatment x sesson interaction	1.970	0.129	3, 58				
**S8**	**F**	**Treatment vs session (Blink amplitude)**	**Tone 2 (Non-paired)**	**Repeated-measures mixed model**	**F value**	**p value**		Geisser-Greenhouse	13, 11		
				Treatment	0.122	0.731	1,22				
				Session	0.186	0.870	2.443,47.23				
				Treatment x sesson interaction	2.741	0.051	3, 58				
**S8**	**G**	**Treatment vs session (Blink amplitude)**	**Tone 3 (Post-paired)**	**Repeated-measures mixed model**	**F value**	**p value**		Geisser-Greenhouse	13, 11		
				Treatment	1.025	0.322	1,22				
				Session	1.697	0.191	2.296, 44.39				
				Treatment x sesson interaction	1.026	0.388	3, 58				
**S8**	**H**	**Treatment vs session (Blink latency)**	**Air puff**	**Repeated-measures mixed model**	**F value**	**p value**		Geisser-Greenhouse	13, 11		
				Treatment	0.411	0.528	1, 22				
				Session	2.193	0.152	1.1, 21.7				
				Treatment x sesson interaction	0.305	0.613	1.1, 21.7				
**S8**	**I**	**Treatment vs session (Blink latency)**	**Tone 1 (Previously-paired)**	**Repeated-measures mixed model**	**F value**	**p value**		Geisser-Greenhouse	13, 11		
				Treatment	0.036	0.852	1, 22				
				Session	0.493	0.679	2.8, 55.1				
				Treatment x sesson interaction	0.655	0.576	2.8, 55.1				
**S8**	**J**	**Treatment vs session (Blink latency)**	**Tone 2 (Non-paired)**	**Repeated-measures mixed model**	**F value**	**p value**		Geisser-Greenhouse	13, 11		
				Treatment	0.958	0.338	1, 22				
				Session	0.617	0.573	2.4, 46.5				
				Treatment x sesson interaction	1.081	0.357	2.4, 46.5				
**S8**	**K**	**Treatment vs session (Blink latency)**	**Tone 3 (Post-paired)**	**Repeated-measures mixed model**	**F value**	**p value**		Geisser-Greenhouse	13, 11		
				Treatment	0.300	0.585	1, 22				
				Session	0.960	0.407	2.6, 69.2				
				Treatment x sesson interaction	0.272	0.817	2.6, 69.2				
**S8**	**J**	**Treatment vs session (Facial motion)**	**All tones**	**Repeated-measures mixed model**	**F value**	**p value**		Geisser-Greenhouse	6, 10		
				Treatment	0.301	0.592	1, 15				
				Session	3.037	0.068	1.855, 27.2				
				Treatment x sesson interaction	1.714	0.201	1.855, 27.2				
**S8**	**K**	**Treatment vs session (Facial motion)**	**Air puff**	**Repeated-measures mixed model**	**F value**	**p value**		Geisser-Greenhouse	6, 10		
				Treatment	3.573	0.078	1, 15				
				Session	2.005	0.146	2.259, 33.13				
				Treatment x sesson interaction	2.613	0.063	3,44				
**S8**	**L**	**Treatment vs session (Facial motion vs Peak spike rate)**	**All tones**	**Repeated-measures mixed model**	**F value**	**p value**		Geisser-Greenhouse	6, 10		
				Treatment	1.636	0.222	2.384, 32.58				
				Session	1.736	0.187	1,14				
				Treatment x sesson interaction	0.349	0.745	2.384, 32.58				
**S8**	**M**	**Treatment vs session (Facial motion vs Peak spike rate)**	**Air puff**	**Repeated-measures mixed model**	**F value**	**p value**		Geisser-Greenhouse	6, 10		
				Treatment	0.098	0.759	2.345, 32.04				
				Session	1.002	0.389	1,14				
				Treatment x sesson interaction	1.033	0.377	2.345, 32.04				
				Treatment	0.957	0.339	1, 21				
				Session	0.893	0.378	1.27, 24.6				
				Treatment x sesson interaction	2.402	0.064	9, 175				
