# Supplementary material for: Psilocybin Attenuates Cortical Representations of Aversion in the Mouse Auditory Cortex
Source: bioRxiv. 2026 Mar 27:2026.03.26.714498. Preprint. [Version 1] doi: 10.64898/2026.03.26.714498 (PMC13041856; doi:10.64898/2026.03.26.714498)
Supplement: Supplement 3 [file media-3.pdf]

Key resources table

Reagents and Biological Resources	Source	Identifier
AAV-syn-GCaMP8f (pGP-AAV-syn-jGCaMP8f-WPRE)	Addgene	# 162376
Dexamethasone 21-phosphate disodium	Sigma-Aldrich (or equivalent)	# 2392-39-4
Xylazine		
Psilocybin (Stock (1 mg/ml in saline))	Cayman	#14041
Artificial cerebrospinal fluid (aCSF)	Prepared in-house	N/A
Ophthalmic eye ointment	Dechra	N/A
Dental cement, Puralube	Yates Motloid	# 44115
Mounting medium with DAPI	Southern Biotech	# 0100-20
Cyanoacrylate tissue adhesive (Vetbond)	3M	#1469SB
Cyanoacrylate tissue adhesive	Krazy Glue	KG58548R
Ultrasound Gel	Aquasonic	# 03-50

Experimental Models: Organisms/Strains

Experimental Models	Source	Identifier
Mouse: *C57BL/6J*	The Jackson Laboratory	Stock #000664

Equipment	Source	Identifier
Small animal stereotaxic frame	David Kopf Instruments	Model 940 (or equivalent)
Two-photon microscope	Bruker	Ultima / Investigator platform
Ti:Sapphire laser (Chameleon Vision S)	Coherent	N/A
25× water-immersion objective (NA 1.05)	Olympus	XLPLN25XWMP2
Orbital nosepiece	Bruker	N/A
GaAsP photomultiplier tubes	Hamamatsu	N/A
Floating Styrofoam ball treadmill and stimuli systems	PhenoSys	JetBall
Infrared camera	Basler	acA1300-60gmNIR
Infrared LED array (850 nm)	Thorlabs	LIU850A
Ultrasonic audio interface	Avisoft Bioacoustics	UltraSoundGate 116H
UV-curable optical adhesive (NOA 71)	Norland Products	NOA 71
Portable LED UV Light Source	U-VIX corporation	# UVC-100
Round glass coverslips (3 mm, 4 mm)	Warner Instruments	# 64-0720/# 64-0724
Hamilton syringe 701RN	Hamilton	# CAL 80330
Hamilton Adaptor	Hamilton	# 55750-01
Glass Capillaries	Harvard Apparatus	# 30-0035
Sugi sponge	Questalpha	#18105-04

Software	Source	Identifier
MATLAB	MathWorks	https://www.mathworks.com
ImageJ / Fiji	NIH	https://imagej.nih.gov
Prism 8 or 10	GraphPad Software	https://www.graphpad.com
R	R Core Team	https://www.r-project.org
Rstudio (2026.01.1)	Posit	https://posit.co
Anaconda	Anaconda Inc.	https://www.anaconda.com
Suite2p	Pachitariu et al., 2017	https://github.com/MouseLand/suite2p
DeepLabCut	Nath et al., 2019	https://github.com/DeepLabCut/DeepLabCut
Prairie view	Bruker	
Phenosys Control and VR	Phenosys	

Code	Source	Identifier
Custom MATLAB analysis scripts	This study	Available upon request
